# Urban–rural disparities in colorectal cancer screening: cross-sectional analysis of 1998–2005 data from the Centers for Disease Control's Behavioral Risk Factor Surveillance Study

**DOI:** 10.1002/cam4.40

**Published:** 2012-10-30

**Authors:** Allison M Cole, J Elizabeth Jackson, Mark Doescher

**Affiliations:** 1 Department of Family Medicine, University of WashingtonSeattle, Washington; 2 Center for Analytics and Public Health, Battelle Memorial InstituteSeattle, Washington

**Keywords:** Cancer prevention, clinical cancer research, screening

## Abstract

Despite the existence of effective screening, colorectal cancer remains the second leading cause of cancer death in the United States. Identification of disparities in colorectal cancer screening will allow for targeted interventions to achieve national goals for screening. The objective of this study was to contrast colorectal cancer screening rates in urban and rural populations in the United States. The study design comprised a cross-sectional study in the United States 1998–2005. Behavioral Risk Factor Surveillance System data from 1998 to 2005 were the method and data source. The primary outcome was self-report up-to-date colorectal cancer screening (fecal occult blood test in last 12 months, flexible sigmoidoscopy in last 5 years, or colonoscopy in last 10 years). Geographic location (urban vs. rural) was used as independent variable. Multivariate analysis controlled for demographic and health characteristics of respondents. After adjustment for demographic and health characteristics, rural residents had lower colorectal cancer screening rates (48%; 95% CI 48, 49%) as compared with urban residents (54%, 95% CI 53, 55%). Remote rural residents had the lowest screening rates overall (45%, 95% CI 43, 46%). From 1998 to 2005, rates of screening by colonoscopy or flexible sigmoidoscopy increased in both urban and rural populations. During the same time, rates of screening by fecal occult blood test decreased in urban populations and increased in rural populations. Persistent disparities in colorectal cancer screening affect rural populations. The types of screening tests used for colorectal cancer screening are different in rural and urban areas. Future research to reduce this disparity should focus on screening methods that are acceptable and feasible in rural areas.

## Introduction

Screening for colorectal cancer has been shown to reduce incidence and mortality associated with the disease by at least 60% [1, 2], yet colorectal cancer remains the second leading cause of cancer death in the United States [3, 4]. At least three options for colorectal cancer screening have been endorsed by the United States Preventive Services Task Force, which does not recommend one screening test over another. For average risk individuals, aged 50–75 years, these options include a highly sensitive stool blood test completed annually, a flexible sigmoidoscopy completed every 5 years, or a colonoscopy completed every 10 years [5]. The 2010 Behavioral Risk Factor Surveillance System (BRFSS) found that age-adjusted screening rates in 50- to 74-year-olds were 11.8% and 65.4% for a stool blood test or sigmoidoscopy or colonoscopy, near the Healthy People 2020 goal of 70.5% screening overall [6].

Although colorectal cancer screening rates have increased over time, increases have been lower in those who are less educated, have a lower income, lack health insurance, and are Hispanic [7, 8]. Analysis of the 1999 and 2008 BRFSS showed that rural residents were also less likely to receive recommended colorectal cancer screening than their urban counterparts [9, 10]. Although the incidence of colorectal cancer in rural areas is similar to urban areas [11], rural residents are more likely to die of the disease [12, 13]. The increased risk of mortality in rural residents may be associated with decreased screening uptake in these areas. Factors affecting rural populations, such as lack of health insurance, increased prevalence poverty, and lower average educational attainment, may contribute to screening disparities for rural populations [9].

Remote rural residents are less likely to be up-to-date on breast and cervical cancer screening [14, 15], a disparity that did not change over the years analyzed (1994–2004). Decreased screening uptake in rural areas may be due to lower rates of health insurance, lower socioeconomic status, and reduced access to care [9]. However, it remains unknown if colorectal cancer screening disparities have persisted over time in rural versus urban areas in the United States. Hence, in this study, our primary aim is to compare urban and rural colorectal cancer screening rates from 1998 to 2005 using the BRFSS, a nationally representative sample. Because of the variety of tests available for colorectal cancer screening, we also aim to understand differences in the types of tests used in rural and urban areas over time. Identification of persistent disparities between the groups would help guide policy efforts and increase funding to increase colorectal cancer screening rates in rural populations.

## Methods

### Sample and subjects

Subjects for this cross-sectional prevalence study came from the national BRFSS, the largest ongoing state-based telephone health surveillance system of noninstitutionalized adults aged 18 years or older in the United States. Additional details about the sampling method, purpose, validity, reliability, and methods of analysis for the BRFSS have been published elsewhere [16, 17]. To assess trends, data from 1998 (*n* = 134,885) to 2005 (*n* = 301,812) were examined. The BRFSS sample size increased during each year examined, but median response rates declined over this time frame; for example, the response rate was 59.1% in 1994 and 51.1% in 2005. Additional information describing the BRFSS data collection process, BRFSS publications, and public use data itself can be accessed at http://www.cdc.gov/brfss/index.htm#about_BRFSS.

### Dependent measures

**Figure 1:**
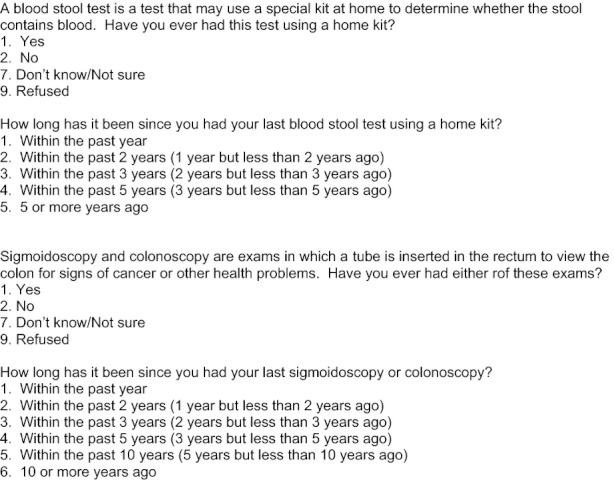
Questions used by BRFSS to assess colorectal cancer screening.

The primary dependent measure was report of timely screening for colorectal cancer for adults age 50 or older, as recommended by the United States Preventive Services Task Force Recommendations [18]. The questions used by BRFSS to assess colorectal cancer screening are seen in Figure 1. We considered respondents up-to-date if they reported receipt of a fecal occult blood test within the preceding 1 year, a flexible sigmoidoscopy within the preceding 5 years, or a colonoscopy within the preceding 5 years. We included respondents who reported being up-to-date with more than one test as being up-to-date in the overall screening outcome, but did not include them in the separate analysis of fecal occult blood testing (FOBT) and endoscopy screening. This was performed to minimize the chances of counting diagnostic tests as screening tests. The format of the questions, which combined up-to-date colonoscopy or up-to-date flexible sigmoidoscopy into one question, prevented us from separating the prevalence of flexible sigmoidoscopy from the prevalence of colonoscopy.

### Independent measures

We ascertained rural residence by classifying respondent's county of residence as being either metropolitan versus nonmetropolitan using Federal Information Processing Standards (FIPS) codes available through the BRFSS. Although the BRFSS is administered in all 50 states, this study excludes states in which no nonmetropolitan counties exist (e.g., New Jersey is excluded because all counties are classified as metropolitan). Also, Alaska was excluded because county-level FIPS codes were not available, which makes differentiation between rural and urban residents impossible for this state. We obtained BRFSS data sets retaining all county-level FIPS codes by written request to the Centers for Disease Control. We broadly grouped counties as metropolitan (urban) or nonmetropolitan (rural) county of residence based on the widely used standard, county-based Office of Management and Budget (OMB) taxonomy. We further subdivided nonmetropolitan counties using the 2003 Urban Influence Code (UIC) groupings of the Economic Research Service of the U.S. Department of Agriculture as follows: “Adjacent Non-Metro” – geographically adjacent to a metropolitan area, including both micropolitan and noncore counties (codes 3–7); “Remote Micropolitan” – not adjacent to a metropolitan county and with a town/urban cluster of 10,000 residents or greater (code 8); and “Remote Non-Core” – not adjacent to a metropolitan county and without a city of 10,000 residents or greater (codes 9–12). UIC adjacency is determined by county boundaries and a minimum work commuting criterion.

### Covariates

Other measures included race/ethnicity (Hispanic, African American, American Indian/Alaska Native, Asian/Pacific Islander, and non-Hispanic white). Because Alaska was excluded from these analyses, the term American Indian is used in lieu of American Indian/Alaska Native, although some BRFSS respondents residing outside of Alaska may, in fact, be Alaska Natives; sex; age (50–64 and 65 years or older); educational attainment (less than high school degree, high school degree or equivalent, or some college); annual household income (less than $25,000; $25,000–$49,999; $50,000–$74,999; and $75,000 or more) and employment status (employed, unemployed, and out of the workforce); general health (poor, fair, good, very good, excellent); marital status (married, divorced, widowed, separated, never married, cohabitating); and health insurance status (yes, no).

### Analytic plan

We evaluated prevalence of colorectal cancer screening overall and prevalence of FOBT or flexible sigmoidoscopy/colonoscopy alone using multivariate logistic regression. We compared colorectal cancer screening rates between urban and rural populations overall and between different categories of rural. We used tests for trends to assess for changes in colorectal cancer screening rates over time. We adjusted all significance tests for the BRFSS's complex sample design using the CDC's formula; we conducted analyses using Stata software (version 11).

## Results

Characteristics of U.S. urban and rural populations in 2005 are described in Table 1. Rural populations are older and have significantly lower education and income levels, lower proportions of minority residents, and a higher proportion of residents reporting fair or poor health as compared with urban populations. A significantly larger proportion of rural populations came from the southeastern United States as compared with urban populations. And a significantly higher proportion of rural residents reported being married as compared with urban residents.

**Table 1:** The characteristics of urban (urban influence code 1–2) and rural (urban influence code >2) populations in the United States, 2005, based on responses to the CDC‘s Behavior Risk Factor Surveillance Survey

Characteristics	Urban (*n* = 311,709)	Rural (*n* = 154,466)
Age^1^		
50–64	54.9%	52.3% (51.8, 52.7%)
65+	45.1%	47.7% (47.3, 48.2%)
Sex		
Male	44.9%	44.9% (44.5, 45.4%)
Race/ethnicity^1^		
Non-Hispanic white	77.6%	88.5%
Black	8.8%	5.2%
Asian	2.1%	0.5%
Native American	0.8%	1.3%
Hispanic	9.3%	3.1%
Other	1.4%	1.5%
Education^1^		
<High school	15.5%	21.0%
High school	55.4%	60.4%
Some college+	29.1%	18.7%
Income^1^		
<$25,000	27.7%	35.8%
$25,000–$49,999	25.8%	28.1%
$50,000–$74,999	12.5%	10.0%
$75,000+	15.6%	7.8%
Missing	18.4%	18.3%
Census region^1^		
Northeast	22.4%	11.7%
Southeast	34.0%	42.8%
Midwest	20.8%	32.4%
West	22.8%	13.1%
Marital status^1^		
Currently married	63.8%	67.5%
General health^1^		
Excellent/good	45.6%	40.3%
Fair	47.2%	49.5%
Poor	7.2%	10.2%

1 *P* < 0.05.

In Table 2, the prevalence of up-to-date colorectal cancer screening in 2005 is shown. Urban areas have higher rates of colorectal cancer screening than rural areas (54.0% vs. 48.3%, *P* < 0.01) even after adjusting for sociodemographic characteristics (age, gender, marital status, education, household income) known to be associated with screening (adjusted rates 54.0% vs. 48.1%, *P* < 0.01). This disparity is largest for remote noncore rural residents, where the adjusted screening rate is 45.2% versus 54.0% in urban areas.

**Table 2:** Unadjusted and adjusted^1^ prevalence of up-to-date colorectal cancer screening^2^ in urban and rural areas of the United States in 2005

	Urban	Rural: Overall	Rural: Adjacent nonmetropolitan	Rural: Remote micropolitan	Rural: Remote noncore
Percent of adults age 50 and older who are up-to-date on CRC screening in 2005	54.0%	48.3%^3^	49.2%^3^	48.5%^3^	44.9%^3^
Adjusted^1^ percent of adults age 50 and older who are up-to-date on CRC screening in 2005	54.0%	48.1%^3^	49.1%^3^	47.8%^3^	45.2%^3^

1 Adjusted for age, gender, race, income, education, general health, employment status, and marital status.

2 Up-to-date defined as fecal occult blood test in last 12 months, flexible sigmoidoscopy in last 5 years or colonoscopy in last 10 years.

3 *P* < 0.05 for comparison to urban screening rate.

**Figure 2:**
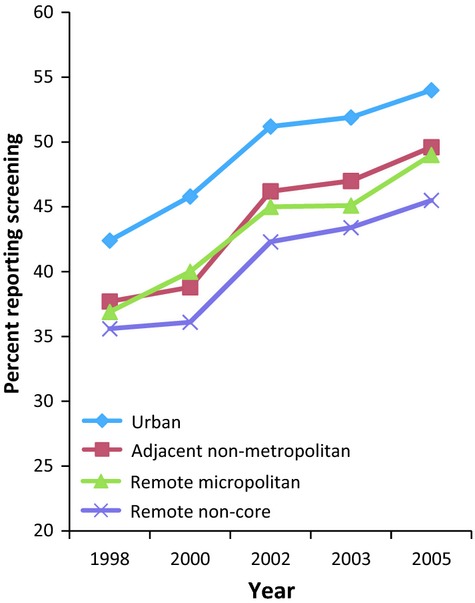
Adjusted* prevalence of up-to-date** colorectal cancer screening in urban and rural areas of the United States 1998–2005. *Adjusted for age, gender, race, income, education, general health, employment status, marital status. **Up-to-date defined as fecal occult blood test in last 12 months, flexible sigmoidoscopy in last 5 years or colonoscopy in last 10 years.

To visualize temporal trends in disparities in colorectal cancer screening, the adjusted prevalence of up-to-date colorectal cancer screening in urban and rural populations from 1998 to 2005 is depicted in Figure 2. The prevalence of up-to-date screening increased in all areas, both urban and rural (test for trend, *P* < 0.001). However, the disparity in colorectal cancer screening rates appears to have persisted throughout the time of this study. The largest disparity is between urban and remote rural populations.

**Figure 3:**
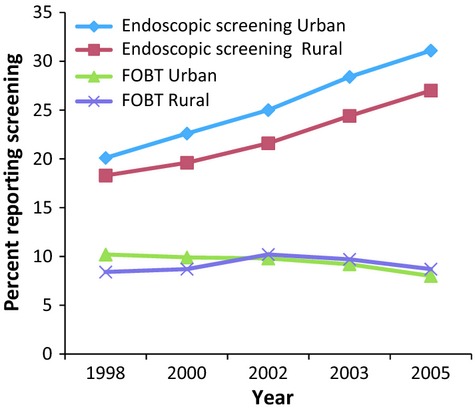
Adjusted* prevalence of up-to-date** colorectal cancer screening using endoscopic testing (flexible sigmoidoscopy or colonoscopy) or fecal occult blood testing (FOBT) in urban and rural populations in the United States, 1998–2005. *Adjusted for age, gender, race, income, education, general health, employment status, marital status. ** Up-to-date defined as fecal occult blood test in last 12 months, flexible sigmoidoscopy in last 5 years or colonoscopy in last 10 years.

We compared the adjusted prevalence of invasive (flexible sigmoidoscopy and colonoscopy) and noninvasive (fecal occult blood test) screening for colorectal cancer screening in both urban and rural populations from 1998 to 2005 in Figure 3. Over this time period, the rate of FOBT decreased in urban populations (test for trend, *P* < 0.001) and increased slightly in rural populations (test for trend, *P* = 0.001). However, the rates of invasive testing increased for both urban and rural populations over the same time period (*P* < 0.001 for both groups). The disparity between urban and rural populations in invasive screening is similar to the overall disparity in screening between urban and rural populations.

## Discussion

Our primary finding is that rural-residing adults in the United States face significant and persistent disparities in colorectal cancer screening compared with their urban counterparts. This disparity is largest in remote rural areas, where the screening rate is roughly 17% lower than urban areas. Although overall colorectal cancer screening rates increased during the time of our study, screening rates for both urban and rural populations remained well below the target of 70.5% outlined in Healthy People 2020. Because screening has been shown to reduce colorectal cancer morbidity and mortality [19–23], missed screening opportunities may lead to poorer colorectal cancer outcomes.

This rural disparity in colorectal cancer screening may be a result of several factors affecting rural populations. Distance barriers, financial barriers, such as high rates of uninsurance, and lack of physicians in many locations all reduce rural residents access to primary care – one of the strongest predictors of up-to-date screening status [24, 25]. Additionally, one screening modality, colonoscopy, typically requires access to specialist physician care, namely gastroenterologists, general surgeons, or gastrointestinal surgeons. Access to all of these specialist types is lower in rural areas compared with urban areas [26]. Thus, efforts to increase colorectal cancer screening in rural areas may need to focus on access to both primary and specialty care.

Evidence gathered by the United States Preventive Services Task Force suggests that all three recommended screening strategies result in similar reductions in colorectal cancer incidence and mortality [2]. To better understand the use of different types of screening tests in rural and urban areas, we separated noninvasive testing (fecal occult blood test) from invasive screening (flexible sigmoidoscopy and colonoscopy) and observed that lower uptake of invasive screening by rural adults explains much of the persistent rural–urban gap in colorectal cancer screening. Previous research has suggested that increases in overall colorectal cancer screening rates nationally are mostly attributable to increase in colonoscopy use [27]. However, decreased access to specialty care in rural areas may prevent widespread adoption of this screening method in rural areas. To address this, one option may be to train more rural primary care providers to perform screening colonoscopy. This approach has been shown to be both safe and effective [28]. However, there may be additional barriers to use of colonoscopy in rural areas, and thus, further research is needed to explore this.

In contrast to invasive screening, noninvasive FOBT use increased slightly in the rural population, while declining in the urban population over the study interval. Research at primary care practices has shown that patients have a preference for colorectal cancer screening by FOBT as opposed to invasive screening [29]. Versus urban CRC screening programs, rural programs that provide a physician recommendation to utilize an FOBT, give FOBT education, or provide a FOBT kit from the provider to the patient are highly effective in increasing this screening method [30]. Several studies have indicated that telephone counseling increases compliance for FOBT or fecal immunochemical testing (FIT), even in patients who were originally nonadherent [31–33]. Additionally, in a study of low-income patients, those offered colonoscopy as the only screening method were less likely to adhere to screening recommendations than patients offered stool testing alone or a choice between the two [34]. Thus, efforts to promote greater use of FOBT in rural primary care practice settings may be an effective and acceptable means of achieving the Healthy People 2020 objective. More research in this area is needed.

Our study has several limitations. Our findings are based on self-report of screening behavior. Although the sensitivity and specificity of self-report for colorectal cancer screening is high [35], patients are more likely to underreport previous colonoscopy than previous stool testing. This may lead to underestimation of colonoscopy and flexible sigmoidoscopy rates relative to stool testing rates [35]. Such bias, if present, would actually indicate that the rural–urban gap in colorectal cancer screening is greater than reported here, because urban populations have a higher prevalence of invasive testing. Another limitation of our study is questions proposed to participants ask only if they have had specific test, not the indication for the test. We assume that receipt of the test represented screening. This introduces potential misclassification bias, if diagnostic tests are being counted as screening tests. However, this is likely to affect both rural and urban populations equally, and therefore unlikely to affect the validity of our conclusions. Finally, the survey design combined colonoscopy and flexible sigmoidoscopy into one response, preventing us from separating these two mechanisms of testing. Because flexible sigmoidoscopy is often offered in a primary care office, and colonoscopy usually requires specialty care, the use of these two services may differ between rural and urban areas, but cannot be ascertained here.

Despite these limitations, our study highlights persistent disparities in colorectal cancer screening for rural residents of the United States. As compared with urban residents, rural residents had persistently lower colorectal cancer screening rates in the 7 years of this study, despite increasing screening rates overall. Remote rural residents consistently had the lowest screening rates of the groups studied. Clearly, efforts to eliminate this gap and achieve the Healthy People target in rural areas are needed. Furthermore, exploration of patient and system level factors that may be contributing to the measured disparities is important in developing programs to improve colorectal cancer screening in rural populations.
